# Exploring the Relationship between Neutrophil Activation and Different States of Canine *L. infantum* Infection: Nitroblue Tetrazolium Test and IFN-γ

**DOI:** 10.3390/vetsci10090572

**Published:** 2023-09-13

**Authors:** Carles Blasi-Brugué, Icíar Martínez-Flórez, Marta Baxarias, Joan del Rio-Velasco, Laia Solano-Gallego

**Affiliations:** Departament de Medicina i Cirurgia Animals, Universitat Autònoma de Barcelona, 08193 Bellaterra, Spain; carles.blasi@uab.cat (C.B.-B.); imflorez.vet@gmail.com (I.M.-F.); martabaxcan@gmail.com (M.B.); rio.joan@gmail.com (J.d.R.-V.)

**Keywords:** NBT, dog, immunity, interferon-gamma, neutrophil, leishmaniosis, oxidative burst, oxidative metabolism

## Abstract

**Simple Summary:**

This study aimed to understand the role of neutrophils in canine leishmaniosis (CanL) by assessing neutrophil activation and its relationship with different states of *Leishmania infantum* infection and antibody and IFN-γ production. The results showed that sick dogs in stage I-mild disease had significantly higher neutrophil activation compared to healthy seronegative and seropositive dogs and sick dogs in advanced stages (II, III–IV). Healthy seropositive dogs exhibited higher neutrophil activation compared to all other groups except sick dogs in stage I. Dogs in advanced disease stages (II, III–IV) did not show significant differences in neutrophil activation compared to healthy seronegative dogs. Furthermore, dogs in stage I had significantly higher IFN-γ concentrations compared to healthy seronegative and sick dogs in advanced disease stages. Dogs in stage II showed higher IFN-γ concentrations compared to healthy seronegative dogs, while no significant differences were observed in dogs in stage III–IV. Healthy seropositive dogs had elevated IFN-γ concentrations compared to healthy seronegative dogs and dogs in stage III–IV. These findings indicate that neutrophil activation is predominant in dogs with mild disease and healthy seropositive dogs with an association with potent IFN-γ production.

**Abstract:**

This study aimed to investigate the role of neutrophils in canine leishmaniosis by assessing neutrophil activation and its relationship with different states of *L. infantum* infection and antibody and IFN-γ production. Dogs were categorized into five groups: healthy-seronegative (*n* = 25), healthy-seropositive (*n* = 21), LeishVet-stage I (*n* = 25), Leishvet-stage II (*n* = 41), and LeishVet-stage III–IV (*n* = 16). Results of the nitroblue tetrazolium reduction test (NBT) showed significantly higher neutrophil activation in stage I (median:17.17, range: [7.33–31.50]%) compared to in healthy-seronegative (4.10 [1.20–18.00]%), healthy-seropositive (7.65 [3.98–21.74]%), stage II (6.50 [1.50–28.70]%), and stage III–IV (7.50 [3.00–16.75]%) groups (*p* < 0.0001). Healthy-seropositive dogs also displayed higher values than all groups except stage I. Stages II and III–IV did not show significant differences compared to healthy-seronegative. Regarding IFN-γ, stage I dogs had higher concentrations (median:127.90, range: [0–3998.00] pg/mL) than healthy-seronegative (0 [0–109.50] pg/mL) (*p* = 0.0002), stage II (9.00 [0–5086.00] pg/mL) (*p* = 0.045), and stage III–IV (3.50 [80.00–548.80] pg/mL) (*p* = 0.02) dogs. Stage II dogs showed increased IFN-γ compared to healthy-seronegative dogs (*p* = 0.015), while stage III–IV dogs had no significant differences compared to healthy-seronegative dogs (*p* = 0.12). Healthy-seropositive dogs had elevated IFN-γ concentrations compared to healthy-seronegative dogs (*p* = 0.001) and dogs in stage III–IV (*p* = 0.03). In conclusion, neutrophil activation was higher in dogs with mild disease and healthy-seropositive dogs, and a relationship between neutrophil activation and the production of IFN-γ was found.

## 1. Introduction

Canine leishmaniosis (CanL) is a zoonotic vector-borne disease caused by the protozoan parasite *Leishmania infantum* and primarily transmitted through the bite of female Phlebotomine sand flies [1]. It has a worldwide distribution and is considered endemic in regions, such as the Mediterranean basin, Latin America, and southern Asia [1]. Dogs serve as the main reservoir for this infection. *Leishmania infantum* is an obligate intracellular parasite that infects and survives within various myeloid lineage cells, including monocytes, macrophages, dendritic cells, and neutrophils. Its presence in the body has unique implications for the immune system during the course of the infection [2].

Clinical manifestations of CanL can vary widely, ranging from subclinical infection to severe systemic disease [1]. The severity of the disease is classified into four stages: mild disease-LeishVet stage I, characterized by mild clinical signs like papular dermatitis, exhibiting no noteworthy clinicopathological abnormalities, and displaying antibody levels that range from negative to low; moderate disease-LeishVet stage II, characterized by diffuse clinical manifestations, like extensive skin lesions, widespread lymph node enlargement or weight loss, clinicopathological abnormalities without a rise in creatinine concentrations, and antibody levels varying from weakly positive to strongly positive; severe disease-LeishVet stage III, defined by extensive clinical manifestations along with clinical signs linked to the deposition of immune complexes (like uveitis or glomerulonephritis), clinicopathological abnormalities, including either IRIS Stage 1 proteinuric or IRIS Stage 2 chronic kidney disease (CKD), accompanied by antibody levels ranging from moderate to high positivity; and very severe disease-LeishVet stage IV, determined by diffuse clinical signs, clinicopathological abnormalities, and advanced CKD stage 3 or 4, accompanied by antibody levels ranging from moderate to high positivity [1]. The interplay between the host’s innate and adaptive immune responses in response to the parasite infection plays a crucial role in determining the clinical signs and overall outcome of the disease [3,4]. Dogs with a predominant cellular immune response are associated with disease resistance or subclinical forms, while dogs exhibiting a predominantly humoral response are associated with clinical illness [2,5]. Adaptive T-cell immunity has been widely investigated to play a crucial role in disease development and resistance. The T helper 1 (Th1)-mediated immune response, characterized by the secretion of cytokines, such as interferon-gamma (IFN-γ), interleukin-2 (IL-2), and tumor necrosis factor-alpha (TNF-α), is associated with immune protection [4,6]. Conversely, the Th2-mediated immune response leads to the production of cytokines, like interleukin-4 (IL-4), interleukin-10 (IL-10), and transforming growth factor-beta (TGF-β), which are linked to a predominantly humoral immune response and disease progression [4,6]. A balanced response between proinflammatory Th1 CD4+ T cells that inhibit parasite replication and an immune regulatory response mediated by T regulatory 1 (Tr1) cells is crucial for disease control [2]. The former stimulates macrophage activity through IFN-γ production, promoting parasite elimination [7]. However, prolonged exposure to antigens can lead to T cell exhaustion, resulting in reduced T cell proliferation and IFN-γ production. This, in turn, allows for parasite survival and expansion within macrophages [7].

In recent years, growing evidence has highlighted the significant role of the innate immune response in controlling *Leishmania* infection. Among the components of the innate immune system, neutrophils have emerged as key players, particularly in the early stages of *Leishmania* infection [8]. While macrophages are considered the primary host target cells for the replication of *L. infantum*, neutrophils also play a distinct role in the control or progression of leishmaniosis, thanks to their ability to kill parasites and produce interleukins [9,10]. As the first line of defense, neutrophils are the initial cells that migrate to the site of infection and internalize the *Leishmania* promastigote [11,12,13,14]. Upon phagocytosis, neutrophils activate their defense mechanisms, including the production of reactive oxygen species (ROS), which is a crucial antileishmanial mechanism produced in phagocytes (oxidative burst) [15,16,17,18]. The phagocyte’s NADPH oxidase generates the superoxide anion (O_2_^−^), which serves as a precursor to hydrogen peroxide and other ROS [15,16,17,19,20,21]. Subsequently, the neutrophil releases these ROS within the phagosomes containing *L. infantum* amastigotes, effectively killing the parasite [19]. In addition to the oxidative burst, neutrophils that ingest *Leishmania* amastigotes initiate further defensive strategies. These include the secretion of TNF-α and IFN-γ, which aid in stimulating macrophage activation and recruitment to the infection site [22,23] and the release of neutrophil extracellular traps, which also potentially play a significant role in combatting protozoal infections, such as *Leishmania* [24].

However, *Leishmania* amastigotes have the ability to deactivate the oxidative activity of polymorphonuclear leukocytes, allowing them to survive within neutrophils and exploit them as a means to hide from the immune system while silently infecting the final host cell, the macrophage [10,22,25]. Alternatively, if the oxidative burst is successful and limits parasite growth within cells, the infection can be controlled [26,27]. Therefore, the ability or predisposition of neutrophils to activate the oxidative burst can have a significant impact on disease progression or containment.

Interferons (IFNs) are a family of proteins that exhibit strong antiviral and antibacterial effects and play crucial roles in regulating effector cells within both innate and adaptive immune responses [28,29,30,31]. Among these, IFN-γ stands out for having the most diverse and potent effects [32]. While much of the research on IFN-γ has focused on its effects on adaptive immunity, recent studies have revealed its ability to modulate myeloid cell activity, including neutrophils, in diverse ways. These include enhancing NADPH oxidase activity and the oxidative burst, promoting nitric oxide (NO) production, increasing the expression of MHCII proteins, altering cytokine and chemokine production, inhibiting chemotaxis, and suppressing apoptosis [33]. Although these effects have been predominantly observed in vitro based on mature neutrophils, studies suggest that more substantial changes occur when IFN-γ interacts with maturing myeloid cells [34]. Thus, IFN-γ could potentially have an impact on the capacity of neutrophils to eliminate phagocyted *Leishmania* amastigotes and promastigotes by stimulating the cellular immune response and activating phagosomes to induce an oxidative burst. Therefore, a positive relationship within IFN-γ and the oxidative metabolism of neutrophils could be suspected.

The nitroblue tetrazolium reduction test (NBT) is a diagnostic assay that utilizes the ability of neutrophils and macrophages to generate free radicals (oxidative burst) during the process of phagocytosis [35,36]. This test relies on the reduction of nitroblue tetrazolium, a colorless compound, to formazan, a blue compound, by the NADPH oxidase present in activated neutrophils within the phagocytic vacuole. As a result, the cytoplasm of the cells undergoes a color change, indicating the production of ROS [36]. The NBT reaction provides an assessment of the ROS-generating activity in the cytoplasm of cells and has been shown to correlate strongly with the levels of ROS produced during the oxidative burst [37,38]. By employing conventional light microscopy, the rate of NBT can be determined by calculating the percentage of neutrophils with formazan present in their cytoplasm, reflecting the activation status of neutrophils and monocytes in the peripheral blood [35]. The primary objective of this study was to assess the variations in peripheral blood neutrophil ROS generation in dogs with different stages of *L. infantum* infection by means of the NBT. Additionally, we aimed to investigate the potential association between neutrophil ROS generation and IFN-γ concentrations, as well as antibody levels, in dogs with different states of *L. infantum* infection.

## 2. Materials and Methods

All dogs included in this study belonged to volunteer owners who had granted permission to a physical examination and blood collection from their dogs through a signed consent form.

### 2.1. Dogs and Clinical Data

Dogs of different breeds from an endemic area were selected to enter the study. A complete blood count, serum biochemistry profile (at least containing creatinine, alanine aminotransferase, and albumin), urinalysis, serum electrophoresis, and ELISA serology were performed for all dogs, as well as cytology of the lesions in dogs in stage I.

A total of 128 dogs were enrolled in this study and divided into five groups according to the LeishVet staging system [1]: Group 1 included healthy seronegative dogs with no clinical signs or hematological or biochemical abnormalities (*n* = 25); Group 2 included *Leishmania*-seropositive but clinically healthy dogs with no clinical signs, hematological and serum and urinary biochemical parameters within normal limits, and presenting low to medium antibody levels (*n* = 21); Group 3 included dogs with mild disease-LeishVet stage I presenting papular dermatitis as the sole clinical sign [39], the identification of intralesional amastigotes via cytology, hematological, serum, and urinary biochemical parameters within normal limits (including creatinine < 1.4 mg/dL), and presenting negative to low antibody levels (*n* = 25); Group 4 included dogs with moderate disease-LeishVet stage II, including the characteristic clinical signs (diffuse clinical manifestations, like extensive skin lesions, widespread lymph node enlargement, or weight loss among others), hematological and serum biochemical abnormalities with normal creatinine values (<1.4 mg/dL), and medium-to-high antibody levels (*n* = 41); Group 5 included dogs with severe disease-LeishVet stage III and very severe disease-Leishvet stage IV, including the characteristic clinical signs, hematological, biochemical abnormalities (including creatinine values of 1.4–2.8 mg/dL or UPC > 1 for stage III and creatinine values > 2.8 for stage IV or UPC > 5), and presenting positive antibody levels (*n* = 16) [1]. The urine protein creatinine ratio was assessed in 12/25 dogs in stage I and in all dogs in stages II, III, and IV. *Leishmania infantum*-specific antibody levels were assessed in all dogs using an end point ELISA. As for IFN-γ, this test was conducted on most of the dogs, although technical limitations prevented its performance in all cases. IFN-γ measurements were obtained from 10 out of 25 healthy seronegative dogs, 15 out of 21 healthy seropositive dogs, 24 out of 25 stage I dogs, 34 out of 41 stage II dogs, and 13 out of 16 stage III/IV dogs. A follow-up of up to one year was available for all dogs with papular dermatitis without conventional anti-*Leishmania* systemic treatment instituted. Some dogs in this group received local treatment whit either topical meglumine antimoniate as a lotion spray (*n* = 6/25), polyhexamethylene biguanide alone or in combination with Toll like receptor 4 agonist as a spray (*n* = 9/25), or placebo (*n* = 9/25), and one received no treatment at all [39].

### 2.2. Blood Collection

The blood was collected via jugular or cephalic venipuncture and placed in EDTA tubes for the hematologic analysis and the NBT test, into heparin tubes to perform cytokine release whole blood assay, and into serum tubes for the antileishmanial antibody quantification.

### 2.3. Antileishmanial Antibody Quantification via ELISA

Antibody levels against *L. infantum* were assessed using an in-house Enzyme-Linked ImmunoSorbent Assay (ELISA), as outlined in previous studies [3,40]. Dog sera were diluted to a ratio of 1:800 in a phosphate buffer solution (PBS) containing Tween 20 and 1% dry milk. This mixture was then incubated in plates previously coated overnight with sonicated promastigotes of *L. infantum* (MHOM/MON-1/LEM-75) obtained from an infected dog at a concentration of 20 µg/mL, and the incubation took place at 37 °C for 1 h [41]. Following this, the plates underwent a series of washes using PBS-Tween and PBS. Next, Protein A conjugated to horseradish peroxidase (Peroxidase Conjugate Protein A; Merck KGaA, Darmstadt, Germany) was added to the plates at a dilution of 1:30,000 and incubated for 1 h at 37 °C. After another round of washing, the plates were treated with o-phenylenediamine and substrate buffer (SIGMAFAST OPD; Merck KGaA, Darmstadt, Germany). The reaction was terminated using 5 M H_2_SO_4_. Absorbance readings were taken at 492 nm using a spectrophotometer (MB-580 HEALES; Shenzhen Huisong Technology Development Co., Ltd., Shenzhen, China). The quantification of results was performed in terms of ELISA units (EUs), normalized against positive canine serum utilized as a calibrator and set at 100 EU.

The threshold was established at 35 EU [39]. Serum samples were categorized as negative when their value fell below 35 EU. If the result ranged from 35 EU up to but not exceeding 150 EU, it was considered low positive. Similarly, if the result fell between 150 EU and 300 EU, it was categorized as medium positive. Finally, if the value equaled or surpassed 300 EU, it was classified as high positive.

For a more in-depth examination of samples identified as medium or high positive, an ELISA involving a two-fold serial dilution was conducted. This process was initiated at a dilution of 1:800 and continued with an additional 7 to 11 dilutions. Quantification was predicated on arbitrary units (EU) in relation to a calibrator set at 100 EU, which corresponded to an optical density (OD) value of 1 at the 1:800 dilution. The mean values of dilutions displaying an OD approximate to one were chosen for calculating the EU. This computation was executed using the following formula: (sample OD/calibrator OD) × 100 × dilution factor [40].

### 2.4. Nitroblue Tetrazolium Reduction Test

For each EDTA tube, blood was drawn and filled into three hematocrit capillary microtubes (Servoprax, Wiesel, Germany). These microtubes were then subjected to centrifugation at 2910× *g* for 5 min (Fugevet+ GDC005, Nahita International Ltd., London, UK) to isolate the buffy coat. The obtained buffy coat was transferred to an Eppendorf tube, where it was mixed with an equal volume of 0.1% NBT solution (1:1) (N6876, Sigma-Aldrich Co., St. Louis, MO, USA). The mixture was gently agitated and then placed in a heater at 37.5 °C for a duration of 15 min (INB 200, Memmert GmbH + Co. KG, Schwabach, Germany), followed by an additional 15 min at room temperature. Subsequently, two blood smears were prepared from each Eppendorf tube, with 3 µL of NBT-stained blood being placed on each glass slide. These slides were further stained using the Diff-Quik method. To assess the NBT rate, a total of 300 neutrophils displaying a clear morphology were counted on each slide using standard light microscopy. Neutrophils that were aggregated or damaged were excluded from the count. The NBT rate was calculated by determining the percentage of activated neutrophils, characterized by the presence of blue-black formazan deposits, among the total counted neutrophils [42].

Dogs were considered to have an increased NBT rate when the NBT value exceeded the cut-off. This cut-off was calculated by considering the NBT values of the 25 healthy seronegative dogs as the control group. A high sensitivity was desired for this cut-off. The standard deviation was added to the mean value of this group, resulting in a cut-off of 10.80%. With this cut-off, considering the healthy seronegative dogs as negative and the dogs with papular dermatitis as positive, the sensitivity was 92% and the specificity 84%.

### 2.5. Cytokine Release Whole Blood Assay and Determination of Canine IFN-γ

The heparinized cytokine release whole blood assay was conducted following established procedures [3]. To outline the process, the assay involved incubating whole blood separately under three distinct conditions: (i) medium alone (unstimulated); (ii) medium containing *L. infantum* soluble antigen (LSA) at a concentration of 10 µg/mL [43]; and (iii) medium supplemented with the mitogen concanavalin A (ConA, 100 mg, Medicago^®^, Uppsala, Sweden) at a concentration of 10 µg/mL. LSA was obtained through a process involving triple cycles of freezing and thawing of cultured *L. infantum* promastigotes (MHOM/MON-1/LEM-75) suspended at a concentration of 1 × 10^9^ cells/mL in PBS. Subsequently, the supernatant was harvested following centrifugation (8.000× *g*, 20 min, 4 °C), adhering closely to the methodology outlined in Carrillo et al., 2015, albeit with minor adjustments [44]. Following five days of incubation at 37 °C in an environment containing 5% CO_2_, supernatants were obtained through centrifugation at 300× *g* for a duration of 10 min. These supernatants were then collected and stored at −80 °C until the testing phase. The measurement of IFN-γ was carried out for all samples utilizing a commercially available sandwich ELISA (Du-oSet^®^ ELISA by Development System R&DTM, Abingdon, UK) using a canine IFN-γ antibody. A standard curve for canine IFN-γ was established through a computer-generated four-parameter logistic curve-fit using the MyAssays program (http://www.myassays.com/) [3]. Dogs were categorized as IFN-γ producers if the concentration of *L. infantum*-specific IFN-γ exceeded or was equal to 110 pg/mL after accounting for the medium-alone baseline [43].

### 2.6. Statistical Analysis

The statistical analysis of data was performed using GraphPad Prism 8.0.1 for Windows software (GraphPad Software, San Diego, CA, USA).

Several normality tests (Anderson–Darling, D’Agostino and Pearson, Shapiro–Wilk, and Kolmogorov–Smirnov) were performed to assess the normality of the different variables (NBT rate, serological results, and IFN-γ concentration). No variables followed a normal distribution except for the NBT rate in stage I and stage III–IV considering a *p*-value < 0.05 as statistically significant. Therefore, non-parametric tests were used to assess the data, except for the unpaired *t*-test (parametric test) performed between the parameters with a normal distribution. A non-parametric Mann–Whitney *U*-test was used for quantitative variables to compare two groups.

A Kruskal–Wallis test and a Dunn’s multiple comparisons test were used to compare more than two groups when data did not follow a normal distribution. If data followed a normal distribution, an ordinary one-way ANOVA and a Tukey’s multiple comparisons test were performed. The Chi-square test or Fisher’s exact test was performed to determine if an association between categorical variables existed. The Spearman (non-normal distribution) or Pearson (normal distribution) correlation coefficient was used to evaluate if there were correlations among different parameters studied. Results were considered statistically significant at a *p*-value < 0.05.

## 3. Results

### 3.1. Dog Clinical Characteristics and Performed Tests

Dog clinical characteristics and results of performed tests are summarized and displayed in Table 1 and Table 2.

Forty-two percent of dogs were crossbred (*n* = 54), and the other most represented breeds were Beagle (*n* = 10), German shepherd (*n* = 8), and Greyhound (*n* = 5).

### 3.2. Age-Stratified Analysis of NBT Rate in Dogs with Papular Dermatitis (Stage I): Insights from Younger and Older Canine Cohorts

Dogs within the stage I group were further subdivided between dogs younger than one year of age (*n* = 22) and dogs older than one year of age (*n* = 3) for a further statistical analysis. No statistical differences were found when comparing the NBT rate of dogs in both groups (*p* = 0.674).

Additionally, the statistical study comparing the stage I group with the other groups was repeated but only taking in consideration the dogs that were older than 1 year of age, showing similar results to the previous study performed. Stage I (older than 1 year of age) had a significantly higher NBT rate than dogs in the healthy seronegative group (*p* = 0.02), the healthy seropositive group (*p* = 0.011), the stage II group (*p* = 0.002), and the stage III–IV group (*p* < 0.0001).

### 3.3. Follow-Up of Dogs with Papular Dermatitis (Stage I)

Over the following months after diagnosis, the majority of the dogs exhibited a notable improvement or the resolution of their cutaneous lesions, and the antibody levels demonstrated a declining trend. However, among the 25 dogs studied, three exhibited a deterioration in their cutaneous lesions, which progressed to the point of ulceration in two cases. Consequently, a systemic conventional anti-*Leishmania* treatment approach was initiated to address their condition.

For the purpose of conducting a statistical analysis, the three dogs that experienced a worsening of their clinical signs were categorized as belonging to group PD-B, and the remaining 22 dogs that displayed an improvement or the resolution of their clinical signs without the need for systemic treatment were designated as members of group PD-A. Detailed results pertaining to NBT, IFN-γ, and serology, both for these delineated groups and for each individual dog within group PD-B, can be found in Table 3.

Upon conducting a comparative analysis of the NBT, IFN-γ, and serology results between the two groups, no statistically significant differences were found (*p* = 0.074, *p* = 0.176, and *p* = 0.271, respectively). However, it is worth noting that the sample size for group PD-B was limited to only three dogs, which could have influenced the robustness of the statistical outcomes.

Interestingly, despite the limitations in sample size, a significant correlation was established between the number of dogs surpassing the NBT cut-off value and the achievement of a clinical improvement without the requirement for systemic treatment. This observation indicated that dogs in group PD-B (those experiencing worsening clinical signs) were more prone to exhibit NBT results within the normal range, unlike dogs in group PD-A (demonstrating an improvement without systemic treatment). However, no corresponding link was identified between the various groups and the status of being an IFN-γ producer or having a positive serology.

### 3.4. Total Leucocyte Concentration and Differential Leukocyte Concentration

The results from the total leucocyte concentration and the differential leukocyte concentration analysis within the different groups studied are displayed in Table 4. Upon scrutinizing the outcomes across different groups, no statistically significant differences were identified in terms of total leukocyte, neutrophil, and eosinophil concentrations. However, significant differences were observed in relation to the lymphocyte concentration. Specifically, healthy seronegative dogs exhibited notably higher lymphocyte concentrations in comparison to the healthy seropositive dogs (*p* = 0.028), as well as to dogs in stage II (*p* = 0.048) and stage III–IV (*p* = 0.001). Interestingly, no substantial variance was found when comparing the lymphocyte concentration between healthy seronegative dogs and dogs in the stage I group. Furthermore, regarding the lymphocyte concentration within the stage I group, significantly higher values were also recorded in contrast to healthy seropositive dogs (*p* = 0.022), dogs in stage II (*p* = 0.033), and dogs in stage III–IV (*p* = 0.001). Notably, no significant differences were evident when comparing the stage II group with the stage III–IV group.

Regarding the monocyte concentration, statistically significant differences were observed between healthy seropositive dogs and those in stage I (*p* = 0.004), as well as between dogs in stage II and those in stage I (*p* = 0.04). In both cases, the stage I group exhibited markedly higher values.

When exploring potential correlations between the NBT rate and both neutrophil and lymphocyte concentrations, a comprehensive analysis encompassing all of the dogs collectively revealed no significant correlations (*p* = 0.491 and *p* = 0.199, respectively). This pattern persisted when conducting separate evaluations within distinct groups, which included healthy seronegative (*p* = 0.682 and *p* = 0.811, respectively), healthy seropositive (*p* = 0.766 and *p* = 0.144, respectively), stage I (*p* = 0.187 and *p* = 0.507, respectively), stage II (*p* = 0.408 and *p* = 0.494, respectively), and stage III–IV (*p* = 0.625 and *p* = 0.525, respectively) groups.

Regarding the monocyte concentration, a significant negative correlation with the NBT was observed exclusively within the stage I group (*p* = 0.005). In contrast, no such correlations emerged when examining the healthy seronegative (*p* = 0.785), healthy seropositive (*p* = 0.128), stage II (*p* = 0.451), and stage III–IV (*p* = 0.73) groups separately. Similarly, no overall correlation was identified when considering all dogs collectively (*p* = 0.159).

### 3.5. Nitroblue Tetrazolium Reduction Test

**Figure 1:**
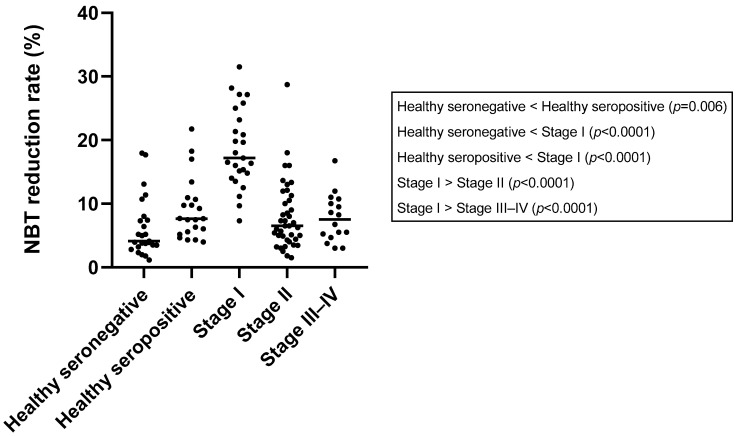
NBT reduction rate results (median, minimum, and maximum values) in different *L. infantum* states of infection.

The results of NBT are depicted in Table 1 and Table 2 and Figure 1. A significantly lower median NBT rate was observed in the healthy seronegative group (median: 4.13% range: [1.15–17.96]%) when comparing this to the to the healthy seropositive group (7.65% [3.98–21.74]%) (*p* = 0.006) and stage I group (17.17% [7.33–31.50]%) (*p* < 0.0001). However, no significant differences were found between the healthy seronegative group and stage II (6.50% [1.50–28.70]%) (*p* = 0.08) or stage III–IV group (7.50% [3.00–16.75]%) (*p* = 0.09).

Regarding healthy seropositive dogs, a significantly lower median NBT rate was also observed when comparing this group to stage I (*p* < 0.0001). However, no significant differences were found with the other groups: stage II (*p* = 0.23) and stage III–IV (*p* = 0.53). A significantly higher median NBT rate was found when comparing stage I to both stage II (*p* < 0.0001) and stage III–IV (*p* < 0.0001). Conversely, there were no significant differences between stage II and stage III–IV (*p* = 0.8).

Regarding the number of dogs over the cut-off value for NBT, there was a significantly higher proportion of dogs that surpassed the cut-off in the stage I group (92.00%) when compared to seronegative (16.00%) (*p* < 0.0001), healthy seropositive (23.8%) (*p* < 0.0001), stage II (24.40%) (*p* < 0.0001), and stage III–IV dogs (18.75%) (*p* < 0.0001). No additional significant differences were found when comparing the other groups (Table 1).

### 3.6. Leishmania Infantum-Specific Antibody Levels

All dogs in the healthy seronegative group were considered negative (median: 5.50, range: [0.30–28.49] EU). Among the healthy seropositive group (median: 133.90, range: [75.79–956.30] EU), 76.19% of dogs were classified as low positive, 14.29% as medium positive, and 9.52% exhibited high antibody levels. In the stage I group (median: 21.42, range: [2.76–227.10] EU), all dogs had negative (76.00%) or low (20.00%) antibody levels, except for one dog (4.00%), which was considered medium positive.

For the stage II group (median: 1894, range: [57.16–10,293.00] EU), 21.95% of dogs were classified as low positive, 34.14% as medium positive, and 43.90% as high positive. In the stage III–IV group (median: 1857, range: [96.79–11,114.00] EU), 37.50% of dogs were classified as medium positive, another 37.50% as high positive, and 25.00% fell into the low positive category.

Statistically significant lower values were found when comparing the healthy seronegative group to the healthy seropositive group (*p* < 0.0001), stage I (*p* = 0.0003), stage II (*p* < 0.0001), and stage III–IV (*p* < 0.0001) groups. The healthy seropositive group showed significantly higher values when compared to stage I (*p* < 0.0001) and significantly lower values when compared to stage II (*p* < 0.0001) and stage III–IV (*p* = 0.0001) groups. Significantly lower values were also observed when comparing stage I to stage II (*p* < 0.0001) and stage III–IV (*p* < 0.0001) groups. No statistically significant differences were observed between the stage II and stage III–IV groups (*p* = 0.93).

### 3.7. IFN-γ Concentration

**Figure 2:**
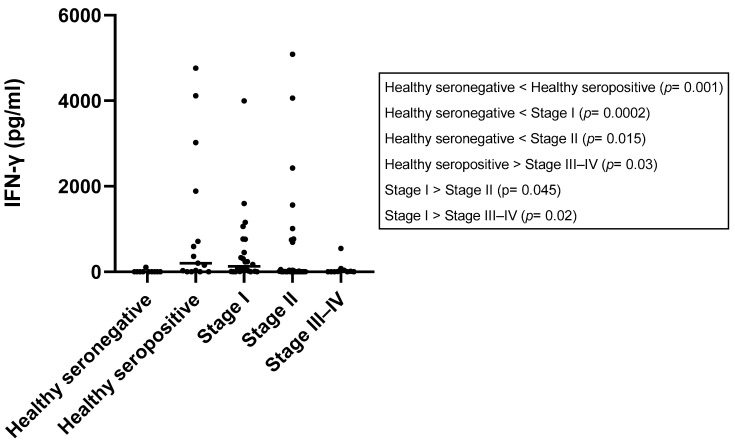
IFN-γ concentrations (median, minimum, and maximum values) after stimulation with LSA in different *L. infantum* states of infection. IFN-y = interferon-gamma. LSA = *L. infantum* soluble antigen.

The results of the IFN-γ concentration are depicted in Table 1 and Table 2 and Figure 2. In the healthy seronegative group, all tested dogs (10/10) were classified as IFN-γ-non-producers. Among the healthy seropositive group, 9 out of 15 dogs (60.00%) were considered as IFN-γ-producers. In the stage I group, 50.00% of dogs (12/24) were classified as IFN-γ-producers. In stage II, a total of 9 out of 34 dogs (26.47%) were classified as IFN-γ-producers, while the majority, 73.53%, were non-producers. Similarly, in stage III–IV, only 4 out of 13 dogs (30.77%) were identified as IFN-γ-producers, with 69.23% classified as non-producers.

Supernatants from LSA-stimulated whole blood of healthy seronegative dogs (median: 0, range: [0–109.50] pg/mL) showed significantly lower concentrations of IFN-γ compared to healthy seropositive dogs (median: 204.10, range: [0–4763.00] pg/mL) (*p* = 0.001), dogs in stage I (median: 127.90 pg/mL, range: [0–3998.00] pg/mL) (*p* = 0.0002), and dogs in stage II (median: 9.00 pg/mL, range: [0–5086.00] pg/mL) (*p* = 0.015). However, no differences were observed when comparing IFN-γ concentration between healthy seronegative dogs and dogs in stage III–IV (median: 3.48 pg/mL, range [0–548.80] pg/mL) (*p* = 0.12).

Regarding healthy seropositive dogs, no significant differences were found when comparing their supernatants from LSA-stimulated whole blood results to dogs in stage I (*p* = 0.6) and stage II (*p* = 0.06). However, statistically significant higher values were observed in healthy seropositive dogs when comparing them to dogs in stage III–IV (*p* = 0.03).

Concentrations of IFN-γ from dogs in stage I were significantly higher than concentrations of IFN-γ from dogs in stage II (*p* = 0.045) and stage III–IV (*p* = 0.02). No statistically significant differences were found between IFN-γ concentrations from dogs in stage II and dogs in stage III–IV (*p* = 0.5).

### 3.8. Correlation between Parameters Studied

No significant correlation was observed between the concentration of IFN-γ after LSA stimulation and *L. infantum*-specific antibody levels (*p* = 0.107) when considering all the groups. Similarly, there was no correlation between the NBT rate and antibody levels (*p* = 0.088). Additionally, no correlation was found between the NBT rate and the concentration of IFN-γ after LSA stimulation (*p* = 0.087).

When examining the correlation within specific groups, no significant correlation was observed between the concentration of IFN-γ and *L. infantum*-specific antibody levels in the healthy seronegative group (*p* = 0.2) and in the stage I group (*p* = 0.146). Conversely, a significant negative correlation was observed between IFN-γ and *L. infantum*-specific antibody levels when evaluating the results in the healthy seropositive (*p* = 0.028), the stage II (*p* = 0.001), and stage III–IV groups (*p* = 0.002).

No significant correlation was found between the NBT results and *L. infantum*-specific antibody levels when comparing different groups, including healthy seronegative dogs (*p* = 0.778), the healthy seropositive group (*p* = 0.938), stage I (*p* = 0.663), stage II (*p* = 0.9), and stage III–IV (*p* = 0.164).

Furthermore, no correlation was observed when comparing the NBT results and the concentration of IFN-γ after LSA stimulation within specific groups, including healthy seronegative dogs (*p* = 0.8), healthy seropositive group (*p* = 0.609), stage I (*p* = 0.94), stage II (*p* = 0.13), and stage III–IV (*p* = 0.16).

**Figure 3:**
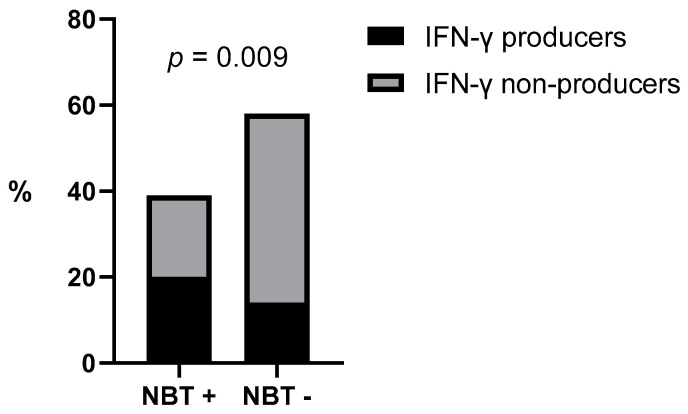
NBT association with IFN-y-producer status. NBT + = increased nitroblue tetrazolium reduction test, NBT − = nitroblue tetrazolium reduction test within normal limits, IFN-y = interferon-gamma.

**Figure 4:**
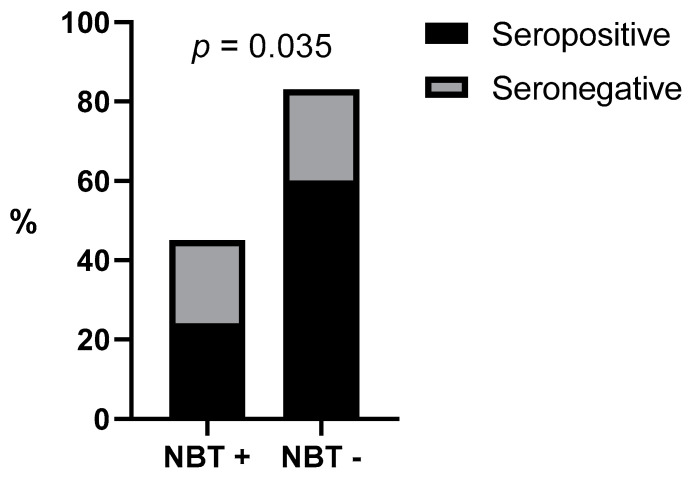
NBT association with the serological status. NBT + = increased nitroblue tetrazolium reduction test, NBT − = nitroblue tetrazolium reduction test within normal limits, ELISA = Enzyme-Linked ImmunoSorbent Assay.

In terms of qualitative values, a significant positive association was found between IFN-γ-producers and increased NBT results when evaluating all dogs (*p* = 0.009) (Figure 3). Additionally, there was a negative association between the proportion of the increased NBT rate and ELISA seropositivity when evaluating all dogs (*p* = 0.035) (Figure 4). A negative association was also found between the IFN-γ-producer status and ELISA positivity (*p* = 0.04). These associations were not found when evaluating the dogs separately by groups.

## 4. Discussion

Neutrophils are suspected to be a key factor for disease development in CanL; however, their role remains poorly understood [2,8]. The objective of this study was to assess neutrophil activation and its relationship with different clinical manifestations ranging from subclinical infection to advanced disease and antibody production and to evaluate if the ability of IFN-γ to stimulate neutrophils correlates with the activation of its oxidative metabolism through NBT measurements. This is the first study to comprehensively evaluate the NBT across all states of this infection, including healthy seropositive and seronegative dogs, and to establish a relationship between antibody levels and the IFN-γ production status.

Regarding the assessment of oxidative metabolism in different clinical stages of leishmaniosis, the present findings revealed that dogs in the mild disease LeishVet stage I exhibited significantly higher NBT values compared to healthy seronegative and seropositive dogs, as well as to dogs in the moderate and severe disease stages. This indicates elevated oxidative metabolism in neutrophils specifically in dogs with mild, self-limiting disease, whereas this increase was not observed in dogs with moderate-to-very severe disease [37]. Additionally, although the healthy seropositive group showed a significantly lower NBT rate than the stage I group, it still displayed significantly higher values compared to all other groups. These results provide support for the hypothesis that neutrophil activation is closely linked to an effective immune response against leishmaniosis. This hypothesis is further supported by the observed association between the lack of an NBT rate increase and the deterioration of clinical signs during the follow-up of dogs with papular dermatitis. It is important to note that among the 25 dogs, only three exhibited worsening clinical signs necessitating systemic treatment, aligning with existing research portraying papular dermatitis as a predominantly self-resolving condition linked to a robust immune response [39,45,46,47]. Remarkably, two of these three dogs did not display an elevated NBT rate, while all dogs manifesting with self-limiting disease (22 out of 25) demonstrated NBT values surpassing the established threshold.

The diminished response observed in the healthy seropositive group, when compared to dogs with mild self-limiting disease (papular dermatitis), could potentially be attributed to different states of infection, resulting in varying levels of immune stimulation or activation. Thus, enhanced oxidative metabolism in neutrophils may serve as an indicator of an effective immune response against *Leishmania* infection, further supporting the significance of neutrophils in disease management.

However, it is important to consider that differences in neutrophil activity within the stage I group could potentially arise from variations in age between these dogs and those belonging to the other groups. Notably, dogs with papular dermatitis are markedly younger than their counterparts, boasting a median age of 6 months, with ages ranging from 3 months to 7 years. This is in line with previous studies, as papular dermatitis is consistently diagnosed in dogs under one year of age [39,48,49]. This has been theorized to be attributed to the condition’s propensity to manifest during the initial encounter between the parasite and the host’s immune system, making it more prevalent in young dogs, particularly in areas where the parasite is endemic [39]. To mitigate the potential bias introduced by age, the statistical analysis was re-executed, focusing exclusively on dogs older than 12 months. This refined analysis yielded outcomes similar to those obtained when considering the entire dog population, despite the limited population of older dogs with papular dermatitis (three cases). Additionally, no significant differences were observed when comparing dogs aged 12 months and above with their younger counterparts within the stage I group. Consequently, the authors consider it unlikely for age to act as a confounding factor in neutrophil activation levels. An alternative hypothesis could be considered, suggesting that the neutrophilic response observed in patients at stage I could be linked to an early phase of the disease rather than a profile of resistance. However, this proposition seems less plausible given that, in the current study, every dog included within the stage I category exclusively displayed papular dermatitis as the solitary clinical manifestation. This particular clinical presentation is commonly associated with a robust and protective immune response. Notably, these dogs spontaneously resolve cutaneous lesions, as evidenced by long-term follow-up, which also indicates an absence of clinical relapse or the emergence of systemic clinical signs, and thus, they are more likely to be representative of an effective immune response and not just an early phase of the disease [39,45,46,47,49]. To further support this, the long-term follow-up in the current study showed that only three animals out of 25 were considered to get worse without systemic treatment, and the other 22 dogs resolved cutaneous lesions and remained healthy. Additionally, as mentioned previously, an association between the lack of an NBT rate increase and the deterioration of clinical signs was found.

In contrast to stage I and healthy seropositive dogs, dogs in stages II and III–IV did not exhibit significant differences compared to healthy seronegative dogs. This observation could indicate a correlation between the absence of neutrophil activation and the progression of clinical disease, mirroring the findings obtained while assessing dogs in stage I who exhibited a deterioration in clinical signs and reflecting a non-effective immune phenotype, including immunity exhaustion [7]. Previous studies have demonstrated an increased rate of neutrophil apoptosis in severe clinical stages of CanL, which may contribute to the reduction in the NBT rate [50]. While an initial increase in oxidative metabolism by neutrophils might be viewed as a protective response, if *Leishmania* infection is not controlled, prolonged neutrophil activation can lead to the generation of oxidative stress and the depletion of antioxidant defenses and ultimately trigger neutrophil apoptosis [51]. In addition, in dogs with LeishVet stage III–IV, chronic kidney disease may also contribute to an increased apoptotic rate and reduced neutrophil superoxide production [52,53]. Regardless of the underlying cause, this negatively impacts neutrophil oxidative metabolism, which can significantly compromise the organism’s primary defense mechanisms. This impairment ultimately results in a reduced ability to control CanL and likely increases the vulnerability to co-infections [50,52,54,55].

When assessing the total leukocyte and leukocyte differential concentrations, despite observing no distinctions in leukocyte, neutrophil, and eosinophil concentrations across the groups, some differences were found when evaluating the lymphocytes and monocytes. Specifically, healthy seropositive dogs and those with moderate-to-advanced leishmaniosis (stage II and stage III–IV) displayed significantly diminished lymphocyte concentrations compared to both healthy seronegative dogs and those in stage I. This partly concurs with earlier research linking clinical systemic CanL to a reduced lymphocyte concentration, consistent with the present observations in stage II and III-V cohorts [56,57]. This could be due to multifactorial mechanisms, and although the most likely explanation is stress-induced changes in the leukogram, other causes, like lymphocyte entrapment or hindered hematopoiesis attributed to bone marrow parasitism, could also contribute to lymphopenia [57]. It is also worth noting that dogs in the stage I group exhibited lymphocyte concentrations comparable to those of healthy seronegative dogs. This observation suggests the absence of a stress leukogram in dogs with mild self-healing disease. Furthermore, dogs in stage I showed a significant increase in the monocyte concentration compared to seropositive dogs and those in stage II. While elevated monocyte concentrations in infected dogs have been documented previously, particularly in dogs with positive splenic cultures for *L. infantum* [58], our study indicated a distinct pattern: solely dogs in stage I exhibited significantly elevated levels, particularly when contrasted against other infection statuses (healthy seropositive and stage II). This could be explained by the increased mobilization of monocytes linked to an effective cellular immune response. Additionally, a correlation between the NBT rate and leukocyte differential was explored, and while none was found when evaluating the neutrophil and lymphocyte concentrations, an inverse correlation surfaced when evaluating the monocyte concentration, though this was only observed within the stage I group. This negative correlation could be explained by the increased recruitment of monocytes to the infection site induced by activated neutrophils, consequently depleting the circulating monocyte pool [22,23].

Previous research has provided evidence for the crucial role of neutrophils in the early stages of *L. infantum* infection [8,11,59]. These cells form an essential part of the initial nonspecific immune response by internalizing the promastigote and acting as a “trojan horse” to infect definitive host cells, such as macrophages [8,11,59]. However, this process could only occur if the parasite is able to evade or suppress the parasite-killing activity of polymorphonuclear cells, which has been observed to happen primarily through inhibiting or evading its oxidative damage capacity [10,59,60]. The findings of the current study support this hypothesis, as dogs with mild self-healing disease and seropositive healthy dogs exhibited a higher rate of NBT in their circulating white blood cells. This indicates increased neutrophil activity and enhanced parasite-killing capacity through the oxidative burst mechanism. This could explain why the infection was confined to the site of inoculation (papular dermatitis) and systemic disease did not develop [39].

Previous studies on neutrophil oxidative metabolism in CanL have reported conflicting results. Some studies suggested a decrease in oxidative metabolism in infected dogs, while others found increased superoxide production and oxidative metabolism [9,36,61,62]. However, these studies had limitations, such as small sample sizes and the lack of disease severity assessments.

Our study findings align with the conclusions drawn in two more recent publications, further demonstrating that neutrophil oxidative metabolism is dependent on the disease stage [36,50]. In line with the study by Gómez-Ochoa et al., our results indicate that dogs with mild disease-LeishVet stage I exhibited significantly higher neutrophil reactivity compared to both healthy dogs and dogs presenting with severe disease [36]. However, in contrast to the findings of Almeida et al., which reported a significantly increased NBT rate in stage II dogs when compared to healthy and stage IV dogs, our study revealed that both stage II and stages III–IV groups exhibited reduced oxidative metabolism, with no significant differences observed between them or the healthy seronegative group [50]. It is important to note that our study included a much larger population, with 41 dogs in stage II (compared to 15 in the previous study), which reduces the risk of population bias. Additionally, we employed a modified NBT technique that mitigates potential biases by evaluating a minimum of 300 cells per sample, whereas previous studies evaluated a minimum of 100 cells per sample, thus enhancing the reliability of our results in this specific aspect [36,50,62]. Furthermore, our study is the first to perform a direct comparison between dogs in stage I and stage II, revealing a significantly higher proportion of activated neutrophils in dogs with mild stage I disease, as well as to evaluate and compare healthy seropositive dogs with both healthy seronegative and diseased dogs. Interestingly, we found a mild yet significant increase in the NBT rate among healthy seropositive dogs when compared to the healthy seronegative group. Finally, this is also the first study to assess the clinical progression of dogs diagnosed with papular dermatitis and compare it to the NBT rate at diagnosis, showing an association of clinical worsening with a lack of neutrophil activation. These findings highlight the distinct neutrophil activation patterns in different states of *L. infantum* infection in dogs and provide valuable insights into the immune responses in CanL.

Gómez-Ochoa et al. proposed a hypothesis suggesting that the variations in neutrophil oxidative metabolism and its augmentation during the early stages of *L. infantum* infection may be associated with the heightened production of chemotactic agents, such as cytokines, in healthy infected dogs, thereby promoting resistance against infection [36]. IFN-γ is a potent immunoregulatory cytokine that not only modulates the adaptive immune system but also plays a role in enhancing neutrophil functions [29,32]. In agreement with this, when evaluating our results, IFN-γ was found to be increased in dogs with mild self-limiting clinical leishmaniosis.

The production of *Leishmania*-specific IFN-γ in stimulated blood, being this a cytokine associated with a robust Th1 immune response, has been previously correlated with a resistant phenotype or a less-severe clinical presentation [2,3,4,63,64,65,66].

Previous studies demonstrated that different clinical stages of leishmaniosis in dogs were associated with different cytokine profiles in stimulated blood [3,66]. They reported that the IFN-γ concentration was significantly higher in dogs staged I and IIa when compared to more severe clinical stages, although results were not compared to healthy dogs [3]. Our study yielded similar findings, as dogs in stage I exhibited significantly higher concentrations of IFN-γ compared to healthy dogs, dogs in the moderate stage I,I, and dogs in the severe stage III–IV disease categories. Notably, dogs in stage II displayed a mild yet significant increase in IFN-γ concentrations compared to healthy seronegative dogs, whereas no significant differences were observed in dogs in stage III–IV when compared to healthy seronegative dogs. Additionally, the majority of dogs in stage II and stage III–IV were classified as IFN-γ-non-producers. Thus, in line with previous studies, dogs in more advanced clinical stages showed lower levels of IFN-γ, which could reflect a less effective immune response, away from a Th1 phenotype [7,67]. It has been previously reported that, as disease progresses, T cells develop certain unresponsiveness to *L. infantum* antigens, showing an impairment in CD4+ T cell proliferation and IFN-γ production, called T-cell exhaustion [7,67].

Interestingly, in our study, healthy seropositive dogs displayed elevated IFN-γ concentrations in comparison to both healthy seronegative dogs and dogs in stage III–IV, the latter of which aligns with a recent study performed in seropositive dogs [40]. However, the previous study did not compare the results to healthy seronegative dogs [40]. This observation could indicate the presence of an effective Th1 response that effectively maintains the infection in a subclinical state.

In a recent study performed on humans, the administration of IFN-γ was found to have notable effects on circulating neutrophils, leading to significant changes in gene expression, protein expression, and overall function. These findings indicate an enhancement in neutrophil activity [32]. The results of that study further suggested that IFN-γ not only stimulates NADPH oxidase activity, thereby promoting the oxidative burst, but also impacts Fc receptor-mediated ingestion, pathogen recognition by innate immune receptors, antigen presentation (including MHCI and MHCII), the activation of GBPs (guanylate-binding proteins), the upregulation of NO production, and the augmentation of neutrophil release into circulation [32]. Therefore, it is plausible that IFN-γ has a direct correlation with NBT by stimulating the oxidative burst of white blood cells, which in turn may be associated with an effective immune response through neutrophil reactivity.

In our study, we discovered a significant association between IFN-γ production and an increased NBT rate. Moreover, both factors exhibited a substantial increase in dogs with subclinical or controlled infection (healthy seropositive), as well as dogs with mild self-limiting disease (stage I). These findings lend support to the notion that IFN-γ plays a role in promoting the oxidative metabolism of neutrophils, thereby contributing to an effective immune response [32]. This suggests that the modulation of neutrophil function through IFN-γ-mediated pathways could serve as one of the mechanisms by which the immune system achieves an efficient defense against the disease [32]. Furthermore, the reduction in IFN-γ associated with T-cell exhaustion could also contribute to the lack of an increase in the oxidative metabolism of neutrophils observed in advanced stages [7,32]. The findings of this investigation also revealed an inverse relationship between IFN-γ and levels of *L. infantum*-specific antibody levels across the healthy seropositive, stage II, and stage III–IV groups. Additionally, an association was found between the IFN-γ-producer status and negative ELISA results. This is in agreement with a previous article by Solano-Gallego et al. published in 2016 [3]. These negative correlations and associations could be explained by IFN-γ’s role as a marker for an effective Th1 response, primarily characterized as cellular rather than humoral. Conversely, heightened antibody levels align with a robust humoral immune response, linked to other cytokines, such as IL-4, IL-10, and TGF-β [4,6,68]. Overall, these findings contribute to a better understanding of the complex immune response in CanL and emphasize the significance of neutrophil activation and IFN-γ production in disease management. Further research is needed to explore other underlying mechanisms and potential therapeutic targets for enhancing the immune response against *Leishmania* infection.

One limitation of this study was the lack of a sufficient number of dogs in stage IV, preventing a separate evaluation of this group. As a result, dogs in stage IV had to be combined with those in stage III in a sole group. However, the classification of dogs into healthy, mild disease, moderate disease, and severe disease categories still holds relevance. Another limitation is inherent to the NBT technique, which relies on a subjective measurement through light microscopy to assess activated neutrophils. To mitigate potential operator bias, a rigorous approach was adopted: a minimum of 300 cells was evaluated per sample, and at least 50% of the samples were independently reviewed by two different investigators.

## 5. Conclusions

In conclusion, neutrophil activation, as assessed using NBT, is significantly higher in dogs with mild self-limiting disease, followed by healthy seropositive dogs, indicating enhanced oxidative metabolism in neutrophils in these cases. Additionally, dogs in stage I exhibiting an NBT rate within normal limits were found to be linked to clinical deterioration when contrasted with dogs displaying an elevated NBT rate at the time of diagnosis. On the other hand, dogs in more advanced stages of the disease did not exhibit significant differences in neutrophil activation compared to healthy seronegative dogs. Furthermore, the study demonstrates a relationship between neutrophil activation and the production of IFN-γ, a key cytokine associated with an effective immune response against *Leishmania* infection. This study provides valuable insights into the role of neutrophil activation and its relationship with subclinical states and degrees of disease severity and suggests that neutrophils and oxidative metabolism could play a crucial role in the effective immune response against leishmaniosis.

## Figures and Tables

**Table 1:** Distribution of qualitative characteristics including breed, sex, positive/negative NBT, positive/negative serology, and productive/non-productive status of IFN-γ among dogs categorized within the five states of infection studied.

Qualitative Characteristics		Healthy Seronegative (*n* = 25)	Healthy Seropositive (*n* = 21)	Stage I (*n* = 25)	Stage II (*n* = 41)	Stage III–IV (*n* = 16)	*p*-Value (Chi-Square, *df*)
**Breed (*n* = 128)**	**Crossbred (*n* = 54)**	9 (36.00%)	11 (52.38%)	14 (56.00%)	16 (39.02%)	4 (25.00%)	0.25 (5.30, 4)
	**Purebred (*n* = 74)**	16 (64.00%)	10 (47.62%)	11 (44.00%)	25 (60.98%)	12 (75.00%)	
**Sex (*n* = 128)**	**Male (*n* = 74)**	12 (48.00%)	10 (47.62%)	16 (64.00%)	23 (56.10%)	13 (81.25%)	0.2 (5.90, 4)
	**Female (*n* = 54)**	13 (52.00%)	11 (52.38%)	9 (36.00%)	18 (43.90%)	3 (18.75%)	
**NBT (*n* = 128)**	**Increased (*n* = 46)**	4 (16.00%)	5 (23.81%)	23 (92.00%)	10 (24.39%)	3 (18.75%)	<0.0001 * (44.60, 4)
	**WNL (*n* = 82)**	21 (84.00%)	16 (76.19%)	2 (8.00%)	31 (75.61%)	13 (81.25%)	
**Serology (*n* = 128)**	**Positive (*n* = 84)**	0 (0.00%)	21 (100.00%)	6 (24.00%)	41 (100.00%)	16 (100.00%)	<0.0001 * (107.80, 4)
	**Negative (*n* = 44)**	25 (100.00%)	0 (0.00%)	19 (76.00%)	0 (0.00%)	0 (0.00%)	
**IFN-γ (*n* = 97)**	**Producers (*n* = 34)**	0 (0.00%)	9 (60.00%)	12 (50.00%)	9 (26.47%)	4 (30.77%)	0.01 * (12.54, 4)
	**Non-prod. (*n* = 63)**	10 (100.00%)	6 (40.00%)	12 (50.00%)	25 (73.53%)	9 (69.23%)	

NBT = nitroblue tetrazolium reduction test, WNL = within normal limits, IFN-γ = interferon-gamma, Non-prod. = non-producers, ELISA = Enzyme-Linked ImmunoSorbent Assay. * Significant differences were observed between groups.

**Table 2:** Median, minimum, and maximum values for quantitative traits, like age, NBT, serology, and IFN-γ within each group.

Quantitative Characteristics Median (Min–Max)	Healthy Seronegative (*n* = 25)	Healthy Seropositive (*n* = 21)	Stage I (*n* = 25)	Stage II (*n* = 41)	Stage III–IV (*n* = 16)	*p*-Value (Kruskal-Wallis Test)
**Age (years)**	1.30 (1.00–1.60)	5.00 (1.00–13.00)	0.50 (0.25–7.00)	4.00 (1.00–13.00)	7.50 (1.00–11.00)	<0.0001 *
**NBT (%)**	4.13 (1.15–17.96)	7.65 (3.98–21.74)	17.17 (7.33–31.50)	6.50 (1.50–28.70)	7.50 (3.00–16.75)	<0.0001 *
**IFN-γ (pg/mL)**	0 (0–109.50)	204.10 (0–4763.00)	127.90 (0–3998.00)	9.00 (0–5086.00)	3.48 (0–548.80)	0.001 *
**Serology (ELISA units)**	5.50 (0.30–28.49)	133.90 (75.79–956.30)	21.42 (2.76–227.10)	1894.00 (57.16–10,293.00)	1857.00 (96.79–11,114.00)	<0.0001 *

NBT = nitroblue tetrazolium reduction test, IFN-γ = interferon-gamma, ELISA = Enzyme-Linked ImmunoSorbent Assay. * Significant differences were observed between groups.

**Table 3:** NBT and IFN-γ results for dogs that presented with papular dermatitis and divided into two groups: Group PD-A (clinical improvement without systemic treatment) and Group PD-B (clinical worsening and need for systemic treatment).

	NBT (%)	NBT Status (Cut-Off = 10.8)	IFN-γ	IFN-γ Status	Serology	Serological Status	Clinical Evolution
**Group PD-A (*n* = 22)**	Median: 17.59 (min: 9.67, max: 31.50)	22/22 Over cutoff	Median: 171.90 (min: 0, max: 3998.00)	10/10 Producers	Median: 18.21 (min: 2.76, max: 227.10)	5/22 positive	Partial or total improvement
**Group PD-B (*n* = 3)**	Median: 8.80 (min: 7.33, max: 20.83)	2/3 WNL	Median: 7.03 (min: 0, max: 234.20)	1/3 Producer	Median: 27.41 (min: 22.34, max: 79.90)	1/3 positive	-
**Dog PD-B.1**	8.80	WNL	7.03	Non-producer	79.90	Positive	Worsening of cutaneous clinical signs: diffuse worsening.
**Dog PD-B.2**	7.33	WNL	234.24	Producer	27.41	Negative	Worsening of cutaneous clinical signs: ulcerative lesion at the nose bridge.
**Dog PD-B.3**	20.83	Over cutoff	0	Non-producer	22.34	Negative	Worsening of cutaneous clinical signs: ulcerative lesion at the level of the right lower eyelid.

Group PD-A = dogs diagnosed with papular dermatitis that showed an improvement in clinical signs without systemic treatment, Group PD-B = dogs diagnosed with papular dermatitis that showed the worsening of clinical signs and in which systemic treatment was considered necessary, NBT = nitroblue tetrazolium reduction test, WNL = within normal limits, IFN-γ = interferon-gamma.

**Table 4:** Median (min–max) results for total leukocyte concentrations and differential leukocyte concentrations in each group.

Median (Min–Max)	Healthy Seronegative	Healthy Seropositive	Stage I	Stage II	Stage III–IV	*p*-Value
**Leucocytes (cell/µL** **)**	11,330 (6390–15,020)	9710 (3360–16,920)	11,180 (6200–20,540)	10,470 (3990–19,430)	9650 (3420–13,630)	0.115
**Neutrophils (cell/µL** **)**	6675 (3706–9913)	5889 (2755–11,844)	6939 (3300–17,860)	7171 (3032–14,378)	6482 (2633–10,223)	0.936
**Lymphocytes (cell/µL** **)**	3006 (1968–3930)	1711 (235–3849)	3590 (900–5800)	1921 (518–4696)	1472 (616–2385)	<0.0001 *
**Eosinophils (cell/µL** **)**	409 (300–934)	514 (63–2369)	500 (20–1100)	479 (0–2176)	386 (0–1204)	0.541
**Monocytes (cell/µL** **)**	668 (313–1502)	413 (34–1157)	750 (100–1310)	517 (129–1269)	560 (171–1204)	0.003 *

* Significant differences were observed between groups.

## Data Availability

Data of the study are available from the corresponding author upon reasonable request.
